# Predators in northern Germany are reservoirs for parasites of One Health concern

**DOI:** 10.1007/s00436-021-07073-3

**Published:** 2021-02-06

**Authors:** Patrick Waindok, Katharina Raue, Miguel L. Grilo, Ursula Siebert, Christina Strube

**Affiliations:** 1 Institute for Parasitology, Centre for Infection Medicine, University of Veterinary Medicine Hannover, Bünteweg 17, 30559 Hanover, Germany; 2 Institute for Terrestrial and Aquatic Wildlife Research, University of Veterinary Medicine Hannover, Hanover, Germany; 3 CIISA—Centre for Interdisciplinary Research in Animal Health, Faculty of Veterinary Medicine, University of Lisbon, Lisbon, Portugal

**Keywords:** Helminths, Zoonoses, Prevalence, *Echinococcus* spp., Red fox, Raccoon dog

## Abstract

Urbanisation and invasion of wildlife into urban areas as well as human leisure activities create diverse wildlife-domestic animal-human interfaces, increasing the risk of (zoonotic) parasite spillover from sylvatic to domestic and synanthropic cycles. This study investigated the endo- and ectoparasite fauna, emphasising on parasites of One Health Concern, of the most common predators in northern Germany between November 2013 and January 2016. Eighty red foxes (*Vulpes vulpes*), 18 stone martens (*Martes foina*) and nine raccoon dogs (*Nyctereutes procyonoides*) were available for the study. Overall, 79 (73.8%) of the examined predators (*n*=107) harboured at least one endoparasite. The most frequently detected endoparasites in red foxes were *Toxocara canis* (43.8% positive individuals), *Capillaria* spp. (36.3%), *Alaria alata* (25.0%), *Echinococcus multilocularis* (26.3%) and *Uncinaria stenocephala* (25.0%). Furthermore, *Toxascaris leonina*, *Trichuris vulpis*, *Taenia* ssp., *Mesocestoides* spp. and coccidian oocysts were observed. The endoparasite species richness in raccoon dogs was comparable to red foxes, while in stone martens, only *Capillaria* spp. were found. Muscle digestion for detection of *Trichinella* spp. and antigen testing for *Giardia* spp. did not show positive results. Ectoparasite analyses revealed infestations with ticks species of the genus *Ixodes* as well as *Dermacentor reticulatus*. Scabies mites were not present in digested skin samples, while *Demodex* spp. mites were observed by faecal flotation in one red fox. Furthermore, fleas (*Archaeopsylla erinacei* and *Chaetopsylla globiceps*) were observed in the fur of red foxes, while lice were not present in any predator species. However, infestation frequency with ectoparasites was with 19.2% generally low in available predator skins (*n*=99). Overall, the present study showed that predators in northern Germany serve as reservoirs for parasites of One Health concern, with four of the five most frequent endoparasites being zoonotic, highlighting the need of parasite surveillance in wildlife predators in order to implement measures avoiding spillovers to domestic animals and humans.

## Introduction

The past and present in central Europe is characterised by a continuous anthropogenic alterations of natural environments, comprising a progressive urbanisation as well as an increasing utilisation of natural habitats for agriculture, forestry or recreational uses (Ellis 2011). However, land conversions inevitably impact resident wildlife species communities, reducing the local biodiversity and/or alter the community composition (Magurran and Henderson 2010; McKinney 2008; Murphy and Romanuk 2014). Furthermore, the fragmentation and the destruction of natural habitats provoke an increasing contact of wildlife with domestic animals, as well as humans, entailing the risk of pathogen spillovers from sylvatic to domestic or synanthropic cycles (Duscher et al. 2015; Hassell et al. 2017). Thus, parasitic diseases of wildlife are of rising One Health concern. Predators, like red foxes (*Vulpes vulpes*), play an important role in transmitting parasites to domestic and synanthropic cycles as. Further common predators at the wildlife-domestic animal-human interface in central Europe are stone martens (*Martes foina*) and raccoon dogs (*Nyctereutes procyonoides*), the latter being one of the most successful invasive carnivores in Europe (Kauhala and Kowalczyk 2011). The emergence of this invading species in northern Germany is illustrated by hunting rates reported by the ministry of the northern German federal state Schleswig-Holstein (Ministry of Energy, Agriculture, the Environment, Nature and Digitalization, abbreviated as MELUND). While the number of hunted foxes and stone martens remained constant since the 1990s, the number of raccoon dogs increased almost exponential since 2004 (MELUND 2019). As in red foxes, the opportunistic feeding habits of raccoon dogs with insects, plants, and small mammals as main food source favours the transmission of parasites, and the presence of various zoonotic parasite species has been documented in both predators, including *Echinococcus multilocularis*, *Trichinella* spp. or *Toxocara canis* (Bruzinskaite-Schmidhalter et al. 2012; Drygala et al. 2013; Ivanov and Semenova 2000; Kornyushin and Malega 2011; Sato et al. 1999; Shimalov and Shimalov 2002; Sutor et al. 2010). Humans can be infected with *E. multilocularis* by accidental ingestion of infective eggs through handling of infected definitive hosts or by oral uptake of contaminated food, water or soil (Deplazes et al. 2011), causing alveolar echinococcosis (AE), a generally fatal disease if left untreated (Brunetti et al. 2010; Pawlowski et al. 2001). Furthermore, humans act as paratenic hosts after accidental ingestion of infective *T. canis* stages, and resulting toxocarosis can have detrimental impacts on health and well-being, ranging from abdominal pain, meningitis and cognitive disorders to irreversible blindness (Auer and Walochnik 2020). Besides endoparasites, native predator species are hosts for a broad range of ectoparasites including ticks and fleas, which play a major role as vectors of various pathogens. In addition, the highly contagious mite *Sarcoptes scabiei* may occur on endemic predator species, particularly red foxes (Soulsbury et al. 2007). The direct as well as indirect contact with infested predators can result in sarcoptic mange of dogs and pseudoscabies in humans (Birk et al. 1999).

Considering the increasing wildlife-domestic animal-human interface, surveillance of parasitic diseases in wildlife plays a crucial role to estimate the potential impact on the concept of One Health. Besides appropriate information of and control measures for humans, knowledge of the parasite fauna in predators provides important information for veterinarians to choose appropriate diagnostic techniques as well as prophylactic and therapeutic strategies to protect domestic animals and their owners from parasitic diseases and break transmission circles. Thus, the aim of the presented study was to investigate the endo- and ectoparasite fauna in the most common terrestrial predators in northern Germany, emphasising on parasites of One Health concern.

## Materials and methods

### Study animals

**Fig. 1:**
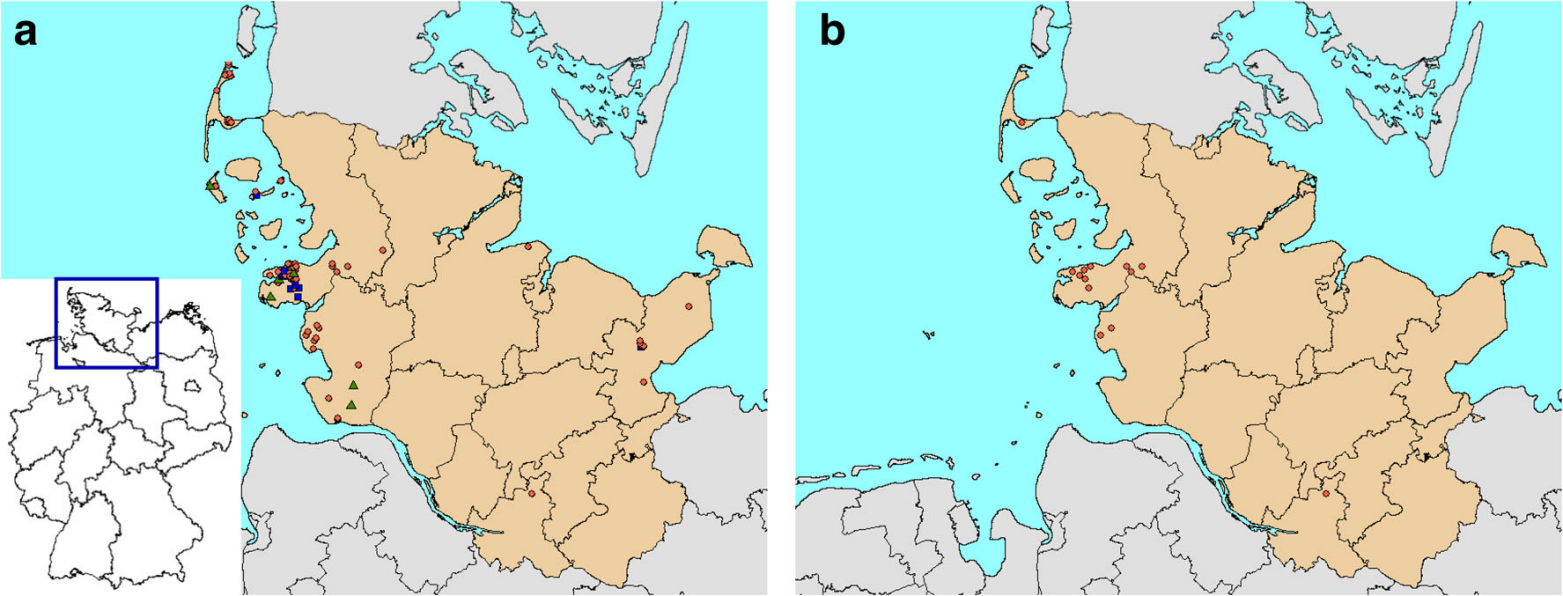
**a** Predator sampling sites in northern Germany. Sampling sites are indicated as follows: red dots, red foxes; blue squares, stone martens; green triangles, raccoon dogs. Origin of eight animals is unknown. The map insert pictures Germany. **b** Detected *Echinococcus multilocularis*-positive red foxes

Examined predators originated mainly from the northern German federal state Schleswig-Holstein (Fig. 1). Sampling locations comprised the districts of Ostholstein, Pinneberg, Rendsburg-Eckernförde, Dithmarschen and North Frisia; including popular tourist destinations like the islands of Sylt and Amrum and the peninsula of Eiderstedt. One specimen originated from the Free and Hanseatic city of Hamburg. The predators were collected from November 2013 to January 2016 and were either shot during legal hunts, found dead or trapped during a project funded by the German Hunting Association (Deutscher Jagdverband) for accreditation of current trapping methods. Trapped animals were anaesthetised by intramuscular injection of 0.05 mg (foxes) or 0.1 mg (stone martens)/kg bodyweight medetomidine hydrochloride (Cepetor®, CP-Pharma, Germany) in combination with 10 mg/kg bodyweight ketamine (Ketamin® 100 mg/ml, CP-Pharma, Germany). For euthanasia, the medicinal product T61® (MSD, Germany) was injected intracardially, containing a combination of embutramide (300 mg/kg bodyweight), mebezonium iodide (75 mg/kg bodyweight) and tetracaine hydrochloride (7.5 mg/kg bodyweight). Raccoon dogs were either shot during legal hunts or found dead.

According to WHO guidelines, carcasses were frozen at −80°C for at least one week before further processing to prevent potential transmission of zoonotic agents, especially *E. multilocularis* (WHO 1984). After thawing carcasses at 4°C, the skin with fur, sections of the intestine and different muscle parts were collected for subsequent parasitological examinations.

### Screening for faecal egg excretion and intestinal parasites

Faecal samples obtained from the distal colon were analysed for parasite eggs and oocysts by the combined sedimentation-flotation technique with ZnSO_4_ (specific gravity 1.3) as flotation medium according to Becker et al. (2016). Additionally, faecal samples were tested for *Giardia* spp. by a commercially available antigen-ELISA (SNAP® Giardia Test, Idexx GmbH).

For determination of adult intestinal parasites, the collected parts of the small and large intestine were opened and the intestinal content was washed through a sieve (mesh size 100 μm) with tap water. The sieve contents were flushed back into a tray and all parasites microscopically visible at × 10 magnification were collected. Furthermore, the intestinal scraping technique was conducted as described by Eckert et al. (2001) to obtain *Echinococcus* spp. and other small helminths. Collected adult parasites and proglottids were identified morphologically to genus/species level based on morphological keys (Macy and Berntzen 1971).

### Screening for *Trichinella* spp. muscle larvae

Predators were analysed for *Trichinella* spp. muscle larvae using pepsin digestion. As the examinations started in 2013, the Commission Regulation (EC) No 2075/2005 (European Commission 2005) was adopted with modifications. Briefly, at least 50 g of tissue derived from skeletal muscles (tongue, masseter, diaphragm and forelimb muscles; approx. 10 g each) were digested in 250 ml of a solution containing 1:100 diluted HCl (37% HCl; Carl Roth GmbH, Germany) and 1% pepsin (Merck, Germany) while stirring at 37 °C until meat particles dissolved (approximately 45 min). Afterwards, the solution was centrifuged at 1500*g* for 5 min. The supernatant was discarded and the pellet resolved in tap water and microscopically examined at 40–100 times magnification for the presence of *Trichinella* spp. larvae.

### Screening for ectoparasites

The whole skin and fur of each specimen was macroscopically examined for the presence of ticks, fleas and lice. In addition, the transport plastic bags were checked for ectoparasites detached during transport or storage. Collected parasites were fixed in 5% formalin (Carl Roth GmbH, Germany) and identified morphologically (Peus 1938; Babos 1964) by microscopical examination.

Analyses for sarcoptic mites were performed using skin digestion methodology. A piece of skin (approx. 1 × 1 cm) from the predator’s head was placed in a 15-ml tube and digested with 5-ml 10% potassium hydroxide (Carl Roth GmbH, Germany) at 37°C for 60 min. Samples were centrifuged at 1500*g* for 10 min and supernatants were discarded. The pellets were suspended in 5-ml 50% glucose solution, centrifuged again at 1500*g* for 10 min and the floats at the surface were transferred to glass slides for microscopical examination at 50–100 times magnification.

## Results

### Parasites detected in the intestine and/or faeces

**Fig. 2:**
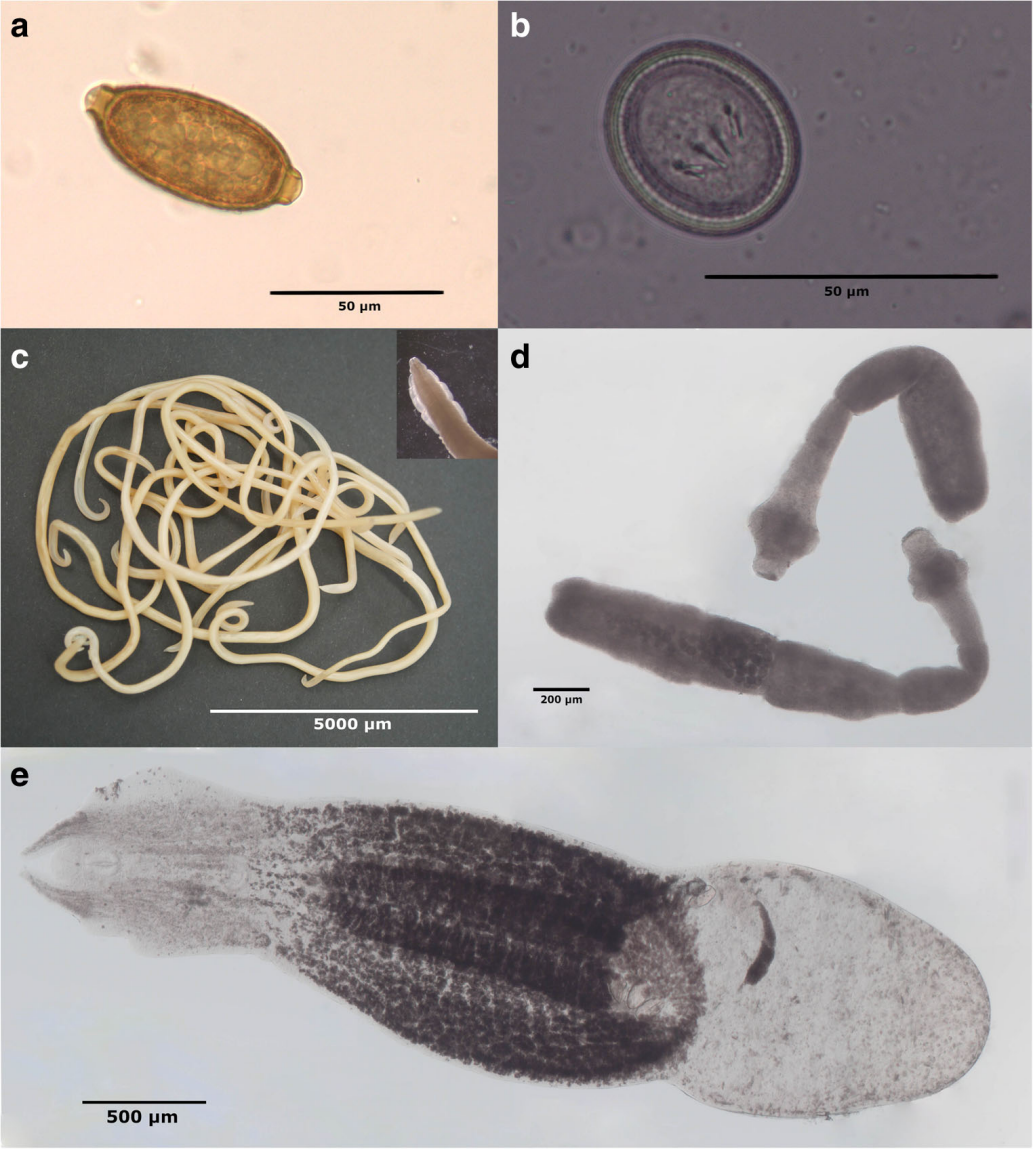
Most frequently detected endoparasites in northern German predators. Eggs of (**a**) *Capillaria* spp. and (**b**) Taeniidae detected in faecal samples; adults of (**c**) *Toxocara canis*, (**d**) *Echinococcus multilocularis* and (**e**) *Alaria alata* detected in the intestines

A total of 107 predators were made available for the analyses, the majority being red foxes (*n*=80; sex: 35 males, 45 females; age: 16 juveniles [deciduous teeth], 60 adults [permanent teeth], 4 unknown), while only 18 stone martens (sex: 7 males, 11 females; age: 3 juveniles, 15 adults) and 9 raccoon dogs (sex: 4 males, 5 females; age: 2 juveniles, 7 adults) were provided. Overall, 79 (73.8%) analysed predators harboured at least one endoparasite. Regarding predator species, 66 (82.5%) red foxes, 6 (33.3%) stone martens and 7 (77.8%) raccoon dogs harboured endoparasites. The endoparasite fauna of the examined predators consisted of at least 9 different species, bearing the potential of greater species richness as detected ancylostomatid, *Capillaria* (Fig. 2a) and taeniid (Fig. 2b) eggs as well as *Taenia* and *Mesocestoides* proglottids, could not be reliably morphologically discriminated to genus and/or species level. Red foxes harboured all of the aforementioned species, while in stone martens only eggs of *Capillaria* spp. were found. As *Capillaria* positivity was based mainly on faecal egg detection in almost all predators, morphological assignment to species level was omitted. Notably, at least seven different endoparasite species occurred in raccoon dogs, although only nine individuals were available for the examination.

The roundworm *T. canis* (Fig. 2c) was the most frequently identified helminth in red foxes (43.8% positive specimens), followed by *Capillaria* spp. (36.3%) and *E. multilocularis* (Fig. 2d) and *Alaria alata* (Figure 2e; 25.0% each). In raccoon dogs, the trematode *A. alata* was the most common helminth (44.4% positive specimens), followed by *T. canis* (33.3%) and taeniid infections (22.2%). As the latter were determined by faecal egg detection, morphological genus differentiation was not possible. Further detected helminths in examined predators were hookworms, *Toxascaris leonina*, *Trichuris vulpis* and *Mesocestoides* species. Regarding protozoans, coproscopic analysis revealed the excretion of coccidian oocysts in 11 (13.8%) predators, while antigen testing for *Giardia* spp. remained negative. Detailed data on observed endoparasites and frequencies are listed in Table 1.

**Table 1:** Endoparasite frequency in terrestrial predators in northern Germany

	Red fox (*n*=80)		Stone marten (*n*=18)		Raccoon dog (*n*=9)		All predators (*n*=107)	
	No.	%	No.	%	No.	%	No.	%
Endoparasite positive	66	82.5%	6	33.3%	7	77.8%	79	73.8%
Endoparasite negative	14	17.5%	12	66.7%	2	22.2%	28	26.2%
Trematoda								
*Alaria alata* (eggs and/or adults)	20	25.0%	0	0.0%	4	44.4%	24	22.4%
Cestodea								
*Taenia* spp. (adults)	13	16.3%	0	0.0%	0	0.0%	13	12.1%
*Echinococcus multilocularis* (adults)	21	26.3%	0	0.0%	0	0.0%	21	19.6%
Taeniidae (eggs)	7	8.8%	0	0.0%	2	22.2%	9	8.4%
*Mesocestoides* spp. (adults)	2	2.5%	0	0.0%	0	0.0%	2	1.9%
Nematoda								
*Uncinaria stenocephala* (adults)	20	25.0%	0	0.0%	1	11.1%	21	19.6%
Ancylostomatidae (eggs)	1	1.3%	0	0.0%	1	11.1%	2	1.9%
*Toxocara canis* (eggs and/or adults)	35	43.8%	0	0.0%	3	33.3%	38	35.5%
*Toxascaris leonina* (eggs and/or adults)	8	10.0%	0	0.0%	1	11.1%	9	8.4%
Ascarids (preadult)	1	1.3%	0	0.0%	0	0.0%	1	0.9%
*Capillaria* spp. (mostly eggs, very rarely adults)	29	36.3%	3	33.3%	1	11.1%	36	33.6%
*Trichuris vulpis* (eggs and/or adults)	8	10.0%	0	0.0%	0	0.0%	8	7.5%
*Trichinella* spp. (muscle larvae)	0	0.0%	0	0.0%	0	0.0%	0	0.0%
Trepomonadea								
*Giardia* spp.	0	0.0%	0	0.0%	0	0.0%	0	0.0%
Coccidia								
Oocysts	11	13.8%	0	0.0%	1	11.1%	12	11.2%

### Co-infections with endoparasites

**Fig. 3:**
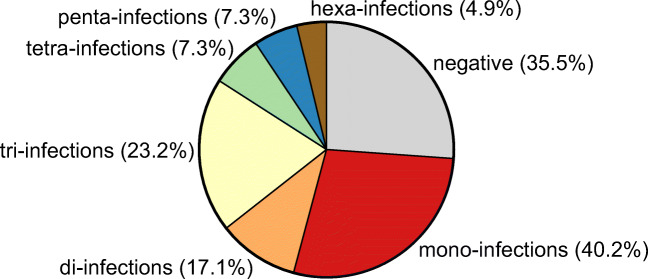
Endoparasite co-infections in northern German predators.

In 30 (38.0%) of the 79 endoparasite positive predators, a mono-infection was determined, while 49 (62.0%) were co-infected with up to six differentiable parasite genera or species, respectively (Fig. 3). Of the positive red foxes, 21 (31.8%) were single infected, while multiple genera/species occurred in 45 (68.2%) of positive individuals. A co-infection with two genera/species was detected in 10 (15.2%), tri-infections in 18 (27.3%), tetra-infections in 7 (10.6%), penta-infections in 6 (9.1%) and hexa-infections in 4 (6.1%) of the endoparasite positive red foxes. The observed co-infections consisted of different combinations including the most frequently detected endoparasites; however, no distinct distribution pattern could be assessed.

In raccoon dogs, three (42.9%) of the endoparasitic positive specimens were mono-infected, while four (57.1%) individuals showed co-infections. Of these, two endoparasite genera/species occurred in one (14.3%) and a tri-infection in three (42.9%) positive raccoon dogs. Similar to red foxes, the most frequently detected endoparasites contributed to the co-infections, but no combination occurred repeatedly.

No co-infection with different endoparasite genera was determined in stone martens as *Capillaria* spp. were the only observed endoparasites in this predator species.

### Screening for *Trichinella* spp. muscle larvae

None of the 100 predators available for *Trichinella* examination (77 red foxes, 18 stone martens and 5 raccoon dogs) showed larvae after artificial muscle digestion, although parts of different muscles were digested to enhance detection of potential infections.

### Infestations with ectoparasites

From 99 predators (76 red foxes, 18 stone martens and 5 raccoon dogs), the skin with fur was available for ectoparasite analyses. Of these, 19 (19.2%) were infested, and seven different ectoparasite taxa were identified. On predator species level, 12 (15.8%) red foxes and 7 (38.9%) stone martens were infested, while no ectoparasites were detected on the five available raccoon dog skins. Ticks of the genus *Ixodes* were the most frequent ectoparasites. Both red foxes and stone martens harboured *Ixodes hexagonus* and *Ixodes canisuga*. While these were the only ectoparasites detected on stone martens, the tick species *Ixodes ricinus* and *Dermacentor reticulates* were additionally recovered from one red fox. Furthermore, infestations with the flea species *Archaeopsylla erinacei* and *Chaetopsylla globiceps* were identified. Additionally, *Demodex* spp. mites were found in one red fox by faecal flotation. Examinations for sarcoptic mites as well as lice did not show positive results*.* Detailed results on the occurrence and frequency of ectoparasite infestations are summarised in Table 2.

**Table 2:** Ectoparasite frequency in terrestrial predators in northern Germany

	Red fox (*n*=76)		Stone marten (*n*=18)		Raccoon dog (*n*=5)		All predators (*n*=99)	
	No.	%	No.	%	No.	%	No.	%
Ectoparasite positive	12	15.8%	7	38.9%	0	0.0%	19	19.2%
Ectoparasite negative	64	84.2%	11	61.1%	5	100%	80	80.8%
Acari								
*Ixodes hexagonus*	4	5.3%	6	33.3%	0	0.0%	10	10.1%
*Ixodes canisuga*	5	6.6%	1	5.6%	0	0.0%	6	6.1%
*Ixodes ricinus*	1	1.3%	0	0.0%	0	0.0%	1	1.0%
*Dermacentor reticulatus*	1	1.3%	0	0.0%	0	0.0%	1	1.0%
*Demodex* spp.*	1	1.3%	0	0.0%	0	0.0%	1	1.0%
Sarcoptic mites	0	0.0%	0	0.0%	0	0.0%	0	0.0%
Insecta								
*Archaeopsylla erinacei*	3	3.9%	0	0.0%	0	0.0%	3	3.0%
*Chaetopsylla globiceps*	1	1.3%	0	0.0%	0	0.0%	1	1.0%
Lice	0	0.0%	0	0.0%	0	0.0%	0	0.0%

*Detected with the combined sedimentation-flotation method

Co-infections of different ectoparasite species were detected in red foxes only. One fox was infested with *I. hexagonus* and *A. erinacei*, and another one with *I. ricinus*, *D. reticulatus* and *A. erinacei*. The red fox identified to be faecal positive for *Demodex* spp. was infested with *I. hexagonus* ticks.

## Discussion

Urbanisation due to increasing urban development and agricultural land use with the consequence of ecological fragmentation as well as the invasion of wildlife into urban areas and sites of people’s leisure activities creates diverse wildlife-domestic animal-human interfaces. Such interfaces represent a critical point for cross-species transmission and emergence of pathogens (Hassell et al. 2017). In the light of the One Health concept, wildlife predators play an important role at these interfaces by maintaining monoxenous or heteroxenous cycles of parasites transmissible to domestic animals or humans. Here, we aimed to identify the parasite fauna in northern German wildlife predators emphasising on parasites of One Health concern. Most predators available for this study were red foxes, which harboured at least nine different endoparasites, reflecting the species diversity recorded in other European countries (Al-Sabi et al. 2013; Borgsteede 1984; Bruzinskaite-Schmidhalter et al. 2012; Criado-Fornelio et al. 2000; Lledo et al. 2015; Smith et al. 2003). Notably, four of the five most frequent endoparasites detected in red foxes are of zoonotic concern.

One of the most important parasitic zoonoses is caused by *E. multilocularis*. Intensive epidemiological research on its endemicity in fox populations since the 1990s showed extensive expansion of this parasite into many European countries (Gottstein et al. 2015), whereby the number of human alveolar echinococcosis (AE) cases correlates with the local abundance of infected red foxes (Schweiger et al. 2007). In southwestern Germany, *E. multilocularis* is highly endemic with about 75% of red foxes being infected, and a human disease incidence of 2.18 cases/100,000 inhabitants in the southwestern federal state of Baden-Wuerttemberg being significantly higher than in Germany as a whole with 0.64 cases/100,000 inhabitants (Schmidberger et al. 2018). However, a northward expansion has been documented in Europe, recognising *E. multilocularis* for the first time in 2000 in Denmark (Saeed et al. 2006) and 2011 in southern Sweden (OIE 2011). In this study, a quarter (26.3%) of available red foxes carried adult *E. multilocularis* in their intestine. Previous studies on the occurrence of *E. multilocularis* in red foxes in the here investigated northern German region remained either negative (Manke 1997) or showed a prevalence of only 0.4% (Nebel 1996). This increase from 0.0–0.4 to 25.3% within 20 years reveals not only newly established endemicity in the most northern part of Germany (cf. Fig. 1b), but may also affect the incidence of human disease. However, between 2001 and 2018, only five human AE cases have been reported in the study area (federal states of Schleswig-Holstein and the Free and Hanseatic city of Hamburg; RKI 2020), and it remained unclear if these infections were autochthonous. Nevertheless, it has to be kept in mind that the actual incidence may be biased since the long incubation period of human AE rather reflects the infection risk 10–15 years ago (Piarroux et al. 2013; Said-Ali et al. 2013; Schweiger et al. 2007). Besides red foxes, the raccoon dog has been identified as an important host for *E. multilocularis* (Oksanen et al. 2016), exhibiting a similar total biotic potential as red foxes (Kapel et al. 2006). Here, *E. multilocularis* was not found in the intestines of examined raccoon dogs; however, only nine specimens were made available for the study.

In recent years, the trematode *A. alata* has gained attention as a One Health threat due to increased findings of its mesocercariae in wild boar meat in Germany, initiating a re-assessment of the potential human health risk (BfR 2007; Möhl et al. 2009; Riehn et al. 2012). While carnivores serve as definitive hosts, mesocercariae can affect a wide range of mammal paratenic hosts including humans and remain infective after transmission between paratenic hosts (Möhl et al. 2009). Here, the frequency of *A. alata* in examined northern German red foxes was 25%. Overall, the *A. alata* prevalence in red foxes in northwestern Europe is wide-ranging with reported values between 17 and 95% in Denmark, Lithuania, the Netherlands and Ireland (Al-Sabi et al. 2013; Bruzinskaite-Schmidhalter et al. 2012; Franssen et al. 2014b; Murphy et al. 2012). Additionally, raccoon dogs are very suitable definitive hosts, showing prevalences of up to 68–97% (Al-Sabi et al. 2013; Bruzinskaite-Schmidhalter et al. 2012; Laurimaa et al. 2016). High susceptibility of this predator species is confirmed by the present study, revealing *A. alata* as the most frequent parasite in raccoon dogs with about half of the examined specimens being infected. However, further research is necessary to estimate the impact of raccoon dogs on *A. alata* transmission cycles and related public health issues in northern Germany as only a low number of specimens were provided for this study.

Nematode meat-transmitted parasites of One Health concern are different species of the genus *Trichinella*. Infections of red foxes with *Trichinella* spp. is considered potential indicator for its occurrence in sylvatic cycles. Consequently, various prevalence studies have been conducted in this predator species (Clausen and Henriksen 1976; Enemark et al. 2000; Hurnikova and Dubinsky 2009; Zimmer et al. 2009). However, the *Trichinella* spp. prevalence has been continuously low in such monitorings (Chmurzynska et al. 2013; Enemark et al. 2000; Franssen et al. 2014a; Zimmer et al. 2009), which is why the absence of *Trichinella* spp. positivity is to be expected if, as in the present study, the number of analysed animals is rather low. Furthermore, freezing of fox carcasses to prevent potential *E. multilocularis* infection of the examiners may influence the detection of *Trichinella* larvae (Franssen et al. 2014a). Therefore, method adjustments are recommended for wildlife meat to improve efficiency (Franssen et al. 2014a).

With an infection rate of 44%, *T. canis*, the zoonotic roundworm of canids, was the most frequent endoparasite in the examined northern German red foxes. Recent studies in countries with comparable climatic conditions report similar prevalences of 59–61% in Denmark (Al-Sabi et al. 2013; Saeed et al. 2006), 41% in Lithuania (Bruzinskaite-Schmidhalter et al. 2012) and 61% in the Netherlands (Franssen et al. 2014b). In the northern German raccoon dogs, the *T. canis* frequency of 33% was lower than that in red foxes. In Denmark and Lithuania, prevalences of 13% and 18% were found (Al-Sabi et al. 2013; Bruzinskaite-Schmidhalter et al. 2012). Interestingly, the present as well as the Danish study (Al-Sabi et al. 2013) found *T. canis* to be more abundant in red foxes and *A. alata* to be more abundant in raccoon dogs. In contrast, in Lithuania only *T. canis* was more abundant in red foxes, while both predators exhibited similar high *A. alata* prevalences of 95% (red foxes) and 97% (raccoon dogs) (Bruzinskaite-Schmidhalter et al. 2012; Franssen et al. 2014b). Although dogs are mostly responsible for environmental contaminations with *T. canis* eggs, red foxes are also important contributors, especially in rural areas (Nijsse et al. 2015). Contaminated soil is considered the main source of human toxocarosis, one of most reported zoonotic helminth infections worldwide (Fakhri et al. 2018; Rubinsky-Elefant et al. 2010). Thus, environmental contamination is a major public health concern as human toxocarosis may cause severe organ injuries, depending on the intensity of infection, the migration behaviour of larvae and the induced immune response (Taylor et al. 1988).

Noteworthy, infections with *Giardia* protozoans, which may be zoonotic depending on the species or genotype, respectively, were not observed in any predator in the present study, although a highly sensitive antigen test was utilised.

As the One Health concept considers not only zoonotic but also pathogens transmissible between wildlife and domestic animals, further endoparasites detected in the northern German predators are reason for concern, especially regarding dogs. Besides *Taenia* and *Mesocestoides* spp. tapeworms, the nematode *Uncinaria stenocephala*, the most common canine hookworm in cooler regions as well as *Toxascaris leonina*, *Trichuris vulpis* and *Capillaria* spp. were observed. As *Capillaria* diagnosis mostly relied on faecal egg detection, morphological species determination was omitted. Thus, it remains unclear whether the predators harboured an intestinal *Capillaria putorii* infection and/or airway infections with *Capillaria aerophila* (syn. *Eucoleus aerophilus)* or *Capillaria boehmi* (syn. *Eucoleus boehmi*). All of them can also infect dogs, and *Capillaria boehmi* has been recently reported as a treatment challenge in parasitised dogs (Gillis-Germitsch et al. 2020). As examined faeces was collected from the distal colon, *Capillaria paranalis* (syn. *Pearsonema paranalis*), a parasite of the anal sac first described in stone martens and recently in red foxes, can be excluded as source of the detected eggs (Forstner and Geisel 1980; Tomczuk et al. 2019*).* Noteworthy, *Capillaria* spp. were the only detected parasites in the examined stone martens. Even though this result is debatable due to low numbers of specimens made available for this study, different previous investigations report rather high *Capillaria* infection rates, but rather low prevelances of other endoparasitic helminths (Gorski et al. 2006; Kornas et al. 2013; Pfeiffer et al. 1989). Finally, it has to be mentioned that the *Capillaria* eggs in the predator’s faces may not result from patent infections but may derive from hunted birds or rodents in terms of an intestinal passage. This also applies to the coccidian oocysts observed in faecal samples of red foxes (14%) and raccoon dogs (11%), as no histological examination of the predator´s intestines was performed.

Besides being hosts for endoparasites of One Health concern, wildlife predators are commonly infested by various species of ectoparasites such as fleas, mites and ticks, and ectoparasites potentially infesting humans or domestic animals may additionally act as vectors for numerous zoonotic pathogens. Overall, ectoparasite detection in the predators was less frequent than expected as only about one fifth of the examined individuals were found to be infested. This may be attributed to active or passive detachment of the parasites during laytime or transport of the carcasses (Lledo et al. 2015), and subsequent freezing at −80 °C may have additionally impeded ectoparasite detection. Furthermore, the rather low numbers of provided predators, especially of stone martens and raccoon dogs, is a general shortcoming of the study. Taking these issues together, the absence of specific ectoparasite species or infestations in general, as observed for raccoon dogs, needs to be interpreted with caution.

In red foxes, the most prevalent ectoparasites were *I. canisuga* and *I. hexagonus*, recorded on 7% and 5% of the specimens, respectively. Both ticks also occurred on stone martens, where infestations with *I. hexagonus* were predominant (33%) compared to *I. canisuga* (6%). Additionally, one red fox was infested with *I. ricinus* and *D. reticulatus*. While *I. canisuga* is not important regarding disease transmission, both *I. ricinus* and *I. hexagonus* are confirmed vectors for spirochetes of the *Borrelia burgdorferi* sensu lato complex and, as well as *D. reticulatus*, for tick-borne encephalitis (TBE) virus. These two pathogens represent the most important tick-borne diseases in Europe, substantially impacting Health systems (Khatchikian et al. 2015; Lohr et al. 2015; Shedrawy et al. 2018). Additionally, dogs and horses may suffer from both pathogens (Klaus et al. 2013; Pfeffer and Dobler 2011; Waldvogel et al. 1981). However, *Anaplasma phagocytophilum* transmitted by *I. ricinus* and *Babesia canis* transmitted by *D. reticulatus* play a more important role in domestic animals in central Europe. Overall, the infestation risk for humans and domestic animals with the two most common tick species in the predators is moderate to low as both *I. hexagonus* and *I. canisuga* are nest-dwelling ticks (Estrada-Peña 2017). Nevertheless, *I. ricinus* infestation in humans and domestic animals and *D. reticulatus* infestation in dogs and horses (Drehmann et al. 2020) are common in Germany, enabling spillover of tick-borne pathogen infections in foxes and other wildlife to humans and domestic animals and thus constituting a One Health issue that should not be neglected. Moreover, predators might not only play a role in the epidemiology of vector-transmitted pathogens by being carriers and disseminator of ectoparasites, but also by serving as pathogen reservoirs, by suppressing the abundance and behaviour of dilution hosts, or, in case of mesopredators like foxes, by suppressing the abundance and behaviour of small predators feeding on rodent reservoir hosts (Haemig et al. 2008). For example, red foxes have been implicated in the spread of TBE since the numbers of red fox correlated positively with the numbers of human TBE cases (Haemig et al. 2008).

Burrowing sarcoptic mites, the etiologic agent of mange, can be transmitted from predators to domestic carnivores, especially those used for hunting, and, occasionally, humans. In this study, infestations with *Sarcoptes scabiei* could not be detected in the examined predators. Noteworthy, sarcoptic mange was also absent in red foxes originating from Danish Jutland, directly bordering to the northern German study region, while 45% of examined red foxes from the region of Copenhagen were infested (Al-Sabi et al. 2014). Interestingly, faecal flotation revealed the presence of *Demodex* spp. mites in one red fox; however, it remains unclear whether this finding resulted from an infestation of the predator or derived from preyed rodents.

Regarding ectoparasitic insects, different flea species have been reported from predatory mammals like red foxes, stone martens or raccoon dogs, including *C. globiceps*, *A. erinacei* and *Ctenocephalides canis* as well as the human flea, *Pulex irritans*, amongst others (Foley et al. 2017; Karbowiak et al. 2016; Kočišová et al. 2006; Vichova et al. 2018). Here, the hedgehog flea, *A. erinacei*, and the fox specialist *C. globiceps* were identified on red foxes, but infestation rates were low with 4% and 1%, respectively. However, it needs to be mentioned that infestation rates and flea species richness in northern German predators is probably higher as fleas start to abandon dead hosts rather quickly (Hsu and Wu 2001). Similar to ticks, fleas not restricted to a certain host species may allow pathogen transmission, e.g. of *Bartonella* spp., from wildlife to (carnivore) domestic animals, and possibly also to humans.

## Conclusions

The endo- and ectoparasite fauna in northern German predators comprises a variety of species, and many of them are of One Health concern. Despite mentioned shortcomings, the results obtained in the presented study can provide valuable information on the potential risk of parasite spillover from predators to humans and domestic animals. Generally, stone martens exhibited the lowest parasite species richness and thus seem to have minor importance in transmissions at the wildlife-domestic animal-human interface, while in the invasive raccoon dog, an endoparasite parasite fauna similar to red foxes has established. Furthermore, the study revealed that *E. multilocularis* is now also being endemic in the most northern part of Germany. Additionally, *A. alata* and *T. canis* were frequently detected zoonotic parasites, but also ectoparasites like ticks may contribute to transmission of pathogens. As the studied predators are widely distributed and urbanisation as well as adaption of predators to anthropogenic surroundings as rural and urban areas enhance the transmission risk to domestic animals and humans, further studies are needed to follow the One Health notion and implement successful surveillance allowing efficient risk assessment of wildlife parasite and vector-borne pathogen spillover.
